# Unexpected Uptake by the Gallbladder in Post-Ablative I-131 Scan

**DOI:** 10.4274/mirt.57441

**Published:** 2015-06-17

**Authors:** Kemal Ünal, Özgür Ümit Akdemir

**Affiliations:** 1 İzmir University Faculty of Medicine, Department of Nuclear Medicine, İzmir, Turkey; 2 Gazi University Faculty of Medicine, Department of Nuclear Medicine, Ankara, Turkey

**Keywords:** Iodine-131, thyroid cancer, gallstone

## Abstract

A 47-year-old woman was diagnosed as papillary thyroid carcinoma. I-131 ablation therapy was applied following total thyroidectomy, and the whole-body scan revealed a focus of increased uptake in the right upper quadrant. Lateral view images of the uptake site showed that the focus was located near the right liver lobe. The patient was referred to radiology department for correlative abdominal Computed Tomography (CT) and Ultrasonography (US) to rule out a possible liver metastasis. CT images detected a gallstone in the corresponding area, which was verified by US. These methods did not reveal any metastatic disease in the liver or in other abdominal organs. This is the first published case report documents a rare false-positive finding of I-131 scan that was associated with an asymptomatic gallstone, and emphasizes the importance of correlative imaging in gallbladder related I-131 uptake.

## Abstract (tr)

Papiller tiroid karsinomu tanısı alan 47 yaşındaki kadın hastaya total tiroidektomi sonrası I-131 ablasyon tedavisi uygulandı. Tedaviden 7 gün sonra yapılan I-131 tüm vücut taraması görüntülerinde, abdomende sağ üst kadranda fokal tarzda yoğun artmış aktivite tutulumu izlendi. Alınan lateral görüntülerde tutulum odağının karaciğer sağ lobu komşuluğuna uyduğu görüldü. Uzak metastatik bir odak hastanın prognozunu değiştireceğinden, beklenmedik bir karaciğer metastazını ekarte etmek ve anatomik korelasyon sağlamak amacıyla hastaya yapılan abdominal Bilgisayarlı Tomografi (BT) ve ultrasonografi tetkiklerinde safra taşı rapor edildi. Her iki incelemede de karaciğerde veya diğer abdominal organlarda metastaz bulgusu saptanmadı. Bu olgu, asemptomatik safra taşına bağlı yalancı pozitif I-131 tutulumunu gösteren ilk bildiri olup, korelatif görüntülemenin önemini de ortaya koymaktadır.

## INTRODUCTION

I-131 ablation therapy is widely used for the treatment of well-differentiated thyroid cancer. Post-ablative scan is a sensitive method for diagnosis of metastatic or residual disease. A false-positive finding of I-131 scan may lead to unnecessary surgery or radio-ablation therapy. The purpose of this study was to emphasize the importance of correlative imaging modalities, especially in patients with suspicion of metastasis.

## CASE REPORT

**Figure 1:**
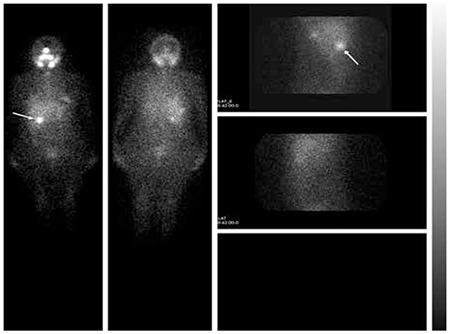
Post-ablative I-131 whole-body scan and lateral view images of the abdomen showed increased uptake in the right upper quadrant

A 47-year-old woman was diagnosed as papillary thyroid carcinoma. Total thyroidectomy was performed in our hospital and I-131 ablation therapy was administered four weeks after surgery. The whole-body scanning was performed on the 7th day of ablation and scintigraphy images revealed a focus of increased uptake in the right upper quadrant. Lateral view images of the uptake site showed that the focus was located in the vicinity of the right liver lobe (Figure 1).

**Figure 2:**
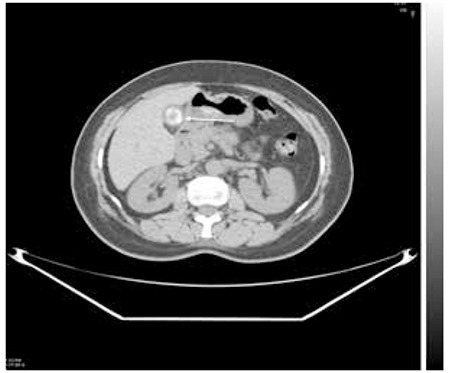
Abdominal Computed Tomography (CT) image with presence of gallstone

The patient was referred to radiology department for correlative abdominal Computed Tomography (CT) and US, to rule out a possible liver metastasis. The CT detected a gallstone in the corresponding area, which was also verified by US (Figure 2). Neither examination identified metastatic disease in the liver or in other abdominal organs.

## LITERATURE REVIEW AND DISCUSSION

The lungs and bone are the most frequent sites of distant metastases in patients with papillary thyroid carcinoma (1,2,3). Liver metastases are not common, especially in low-risk patients. An unexpected distant metastasis changes the prognosis and therapeutic evaluation of the patient.

There are a lot of causes for false-positive findings on I-131 scan other than those related to the gallbladder, such as inflammatory diseases, radioactive contamination, esophageal pathologies, skin burn, pericardial effusion, renal cyst or even non-thyroidal neoplasms (4,5,6). There are only a couple of case reports on the uptake of I-131 by the gallbladder. Chronic cholecystitis and excretion into the biliary tract were previously reported as rare causes of gallbladder uptake (7,8,9). Additional imaging was performed to exclude such conditions, since liver metastasis was not expected in our patient. Low resolution of I-131 images and the requirement of anatomic correlation were the other important reasons for further imaging.

Gallstone related I-131 uptake might be due to recurrent cholecystitis and organification of iodine in leukocytes around the gallbladder wall (10). Although the patient was asymptomatic, biochemical changes might have occurred at the cellular level during the chronic disease course. This biochemical reaction can be verified by histopathological tissue analysis.

This is the first case report that documents a false-positive uptake of I-131 scan that was associated with an asymptomatic gallstone, and it emphasizes the importance of correlative imaging in gallbladder diseases.

## References

[1] Shaha AR, Ferlito A, Rinaldo A (2001). Distant metastases from thyroid and parathyroid cancer. ORL J Otorhinolaryngol Relat Spec.

[2] Mazzaferri EL (1999). An overview of the management of papillary and follicular thyroid carcinoma. Thyroid.

[3] Mazzaferri EL, Massoll N (2002). Management of papillary and follicular (differentiated) thyroid cancer: new paradigms using recombinant human thyrotropin. Endocr Relat Cancer.

[4] Pochis WT, Krasnow AZ, Isitman AT, Cerletty JM, Kir KM, Hellman RS, Collier BD (1990). The radioactive handkerchief sign. A contamination artifact in I-131 imaging for metastatic thyroid carcinoma. Clin Nucl Med.

[5] Bakheet SM, Hammami MM, Powe J (1996). False-positive radioiodine uptake in the abdomen and the pelvis: radioiodine retention in the kidneys and review of the literature. Clin Nucl Med.

[6] Geatti O, Shapiro B, Orsolon PG, Mirolo R, Donna A (1994). An unusual false-positive scan in a patient with pericardial effusion. Clin Nucl Med.

[7] Brucker-Davis F, Reynolds JC, Skarulis MC, Fraker DL, Alexander HR, Weintraub BD, Robbins J (1996). False-positive iodine-131 whole-body scans due to cholecystitis and sebaceous cyst. J Nucl Med.

[8] Seok JW, Kim SJ, Kim IJ, Kim YS, Kim YK (2005). Normal gallbladder visualization during post-ablative iodine-131 scan of thyroid cancer. J Korean Med Sci.

[9] Carlisle M, Cortes A, McDougall IR (1998). Uptake of I-131 in the biliary tract: a potential cause of a false-positive result of scintiscan. Clin Nucl Med.

[10] Siegel E, Sachs BA (1964). In vitro leukocyte uptake of 131-i labeled iodide, thyroxine and triiodothyronine, and its relation to thyroid function. J Clin Endocrinol Metab.

